# All Military Adolescents Are Not the Same: Sexuality and Substance Use among Adolescents in the U.S. Military Healthcare System

**DOI:** 10.1371/journal.pone.0141430

**Published:** 2015-10-29

**Authors:** David A. Klein, William P. Adelman, Amy M. Thompson, Richard G. Shoemaker, Jane Shen-Gunther

**Affiliations:** 1 Department of Family Medicine, Fort Belvoir Community Hospital, Fort Belvoir, VA, United States of America; 2 Department of Pediatrics, Uniformed Services University of the Health Sciences, Bethesda, MD, United States of America; 3 Department of Pediatrics, San Antonio Military Medical Center, San Antonio, TX, United States of America; 4 Clinical Investigations, Fort Belvoir Community Hospital, Fort Belvoir, VA, United States of America; 5 Department of Clinical Investigation and Department of Obstetrics and Gynecology, San Antonio Military Medical Center, San Antonio, TX, United States of America; University of Western Brittany, FRANCE

## Abstract

Data examining sexuality and substance use among active duty and military-dependent youth is limited; however, these psychosocial factors have military implications. Adolescents and young adults aged 12–23 were recruited from an active-duty trainee clinic (n = 225) and a military pediatric clinic (n = 223). Active duty participants were more likely to be older, male, White, previous tobacco users, and report a history of sexual activity and less contraception use at their most recent intercourse, compared to the dependent group. Over 10% of all participants indicated attraction to members of the same gender or both genders. In logistic regression analysis, non-White participants were less likely to use contraception compared to White participants. Adolescents and young adults seen in military clinics frequently engage in high-risk behavior. Clinicians who care for military youth should assess their patient’s psychosocial history. Further study of this population is warranted to identify factors that may influence risk and resilience.

## Introduction

Data examining sexual and substance use risk among active duty and military-dependent youth is limited, and direct clinical comparisons of active duty compared to dependent populations do not currently exist. However, these psychosocial factors have specific military implications, including resourcing health care clinics for these populations.

The United States military is an adolescent-centric organization. Approximately 650,000 active duty service members, or 45% of the total forces in the United States Military are between 17 and 25 years of age; of these, 15% are female[1]. Young service members are known to be at risk for sexually transmitted infections, high-risk sexual behavior (e.g. low rates of contraception use), and substance abuse[2–7]. A large study of active duty military service members in 2011 suggested that 1.3% abused prescriptions drugs, 25% currently smoke, and 11% classify as problematic consumers of alcohol[6].

An additional 500,000 adolescents aged 12–23 receive care each year in the military health system by virtue of their parent’s current or previous affiliation with the United States Armed Services[8]. These adolescents appear to have a unique health risk profile due to their military affiliation, which may include parental deployment and frequent relocation. Existing studies suggest that military-affiliated youth demonstrate resilience, but also high rates of sexually transmitted infection, stress, and substance use[2,9–16]. However, these adolescents appear to have a lower rate of pregnancy compared to their civilian peers[17–18]. The health of this population has direct implications for military readiness (e.g., their family member’s readiness), and the strength of future military recruitment, because military dependent adolescents tend to enlist in the Armed Services at a higher rate than adolescents not affiliated with the military[19].

In 2011, lesbian, gay, and bisexual military service members were permitted to openly serve in the United States Armed Forces. An estimated 50,000 active duty service members at that time were thought to identify as lesbian, gay, or bisexual[20]. Because sexual minority youth are at additional risk for depression, suicidality, sexually transmitted infections, and other health disparities, clinicians caring for such youth should be aware of their patient’s needs[21]. It is possible that the culture in military treatment facilities leads clinic personnel to underestimate the prevalence of sexual minority youth in their care. Similarly, patients and their family members may be less likely to voluntarily disclose such information because of perceptions of military culture. Precise estimates of service members’ sexual attraction have not been studied.

In this study, the authors aim to (1) characterize and compare the sociodemographic and patient profiles of active duty and dependent adolescents in the military healthcare system to prepare clinicians in practice; and (2) explore and elucidate risk factors among youth in the U.S. Military Healthcare System to generate additional research.

## Materials and Methods

This study was reviewed and approved by the institutional review board of Brooke Army Medical Center, Fort Sam Houston, Texas.

### Participants and Procedures

Adolescents and young adults aged 12–23 years were recruited from an active-duty trainee clinic (ages 17–23, n = 225) and a military pediatric clinic (ages 12–23, n = 223) by convenience sampling of consecutive clinic patients over a four-month period in 2013. Both clinics are located in San Antonio, TX, USA and together serve approximately 15,000 patients at any given time. The trainee clinic serves those in advanced training for at least three months who are typically stationed at military bases in the United States and abroad. Military-dependent adolescents are defined as individuals who are eligible for care in the U.S. Military Health System by virtue of their parent’s current or prior affiliation with the Armed Services. Spouses of service-members are not included in this analysis. The military dependent clinic is thought to have a patient demographic representative of the overall military dependent population, with a mix of patients who are sponsored by both active duty and retired service members.

Individuals were approached before they completed their medical visits. Participants under 18 years of age who voiced interest in completing the study provided written assent. Written consent was also obtained from their parents or legal guardians. Those aged 18 and over provided written consent. Patients were excluded from the study if they were unable to provide appropriate consent or assent, or unable to complete the study questionnaire due to a disability or acute illness. Five percent of those approached in the trainee clinic and ten percent of those in the military dependent clinic declined participation; this was typically due to acute illness. Less commonly and exclusively in the military dependent clinic, parents refused to allow their children to participate.

### Measures

Participants completed a study questionnaire regarding sociodemographics and risk behaviors that were modified from a questionnaire used in prior study[22–23]. Participants completed the questionnaires in a room with other study participants and a member of the research team, but did not consult with the research team representative, their parents or legal guardians, or other participants regarding their responses. The research team representative offered procedural help only.

### Data analysis

The demographic data included: age (continuous variable); gender (male or female), race/ethnicity (White, Black, Hispanic, or Other), trouble paying household expenses (yes, no); and attraction (to same gender, opposite gender, or both). Additional questions were targeted at participants’ sexual preferences and practices, contraception use, prior sexually transmitted infections, and substance use and abuse. Of those members who reported prior oral, vaginal, or anal intercourse, questions were posed about contraception and substance use before their last intercourse.

The data was analyzed using SPSS version 20. Descriptive statistics were used to highlight participant characteristics and prevalence data. In bivariate analysis, P values were calculated by the Fisher Exact Test or Pearson’s Chi Square to determine differences between duty status or use of contraception among sociodemographic outcomes of interest. Logistic regression analysis was performed to evaluate contraception use before most recent intercourse and a binarized sociodemographic outcome of interest. Subgroup analyses included participants aged 15–19 years, for purposes of making observations related to the study population in relation to national samples, as well as for ages 17–23 years, for purposes of directly comparing similarly aged active duty participants to dependents. A significance level of 0.05 was used for all statistical tests.

## Results

A total of 448 participants aged 12–23 years enrolled in the study; 47.6% self-reported as male and 50.4% as white. Of these, 9.2% used alcohol at least weekly, 28.1% previously used marijuana, and 8% previously used prescription drugs recreationally. On questioning about sexually transmitted infection, 7.4% reported prior Chlamydia Trachomatis or Neisseria Gonorrhoeae infection. Furthermore, 16.4% of the active duty population and 10.8% of the military dependent population indicated attraction to the same gender or both genders (Table 1). 315 participants reported previous sexual intercourse, of whom: 59% used (or their partner used) prescription contraception, 61.4% used condoms, 81.5% used either method, and 39.3% used both methods at their most recent intercourse (Table 2). Differences between gender and ethnicity were addressed with multivariate analysis (see below).

**Table 1:** Characteristics of participants who presented for care in military adolescent clinics (n = 448).

Variable	Active Duty (n = 225)	Dependent (n = 223)
Age (mean, SD)	20.3 (1.6)	17.2 (2.3)
Gender (n, %)		
Male	129 (57.3%)	84 (37.7%)
Female	96 (42.7%)	139 (62.3%)
Race (n, %)		
White	137 (60.9%)	89 (39.9%)
Black	33 (14.7%)	58 (26.0%)
Hispanic	32 (14.2%)	63 (28.3%)
Other	23 (10.2%)	13 (5.8%)
Trouble paying household expenses (n, %)		
No	102 (45.3%)	153 (68.6%)
Yes	123 (54.7%)	68 (30.5%)
Unknown	0 (0%)	2 (0.9%)
Attracted to (n, %):		
Opposite Sex	185 (82.2%)	199 (89.2%)
Same Sex	24 (10.7%)	9 (4.1%)
Both	13 (5.8%)	15 (6.7%)
Unknown	3 (1.3%)	0 (0%)
Ever had sexual intercourse (n, %)		
Yes	196 (87.1%)	119 (53.4%)
No	29 (12.9%)	104 (46.6%)
Smoked ≥ 100 cigarettes in lifetime (n, %)		
Yes	64 (28.4%)	22 (9.9%)
No	161 (71.6%)	201 (90.1%)
Over past 3 months, consumed alcohol-containing drinks at least weekly (n, %)		
Yes	23 (10.2%)	18 (8.1%)
No	202 (89.8%)	205 (91.9%)
Prior marijuana use (n, %)		
Yes	70 (31.1%)	56 (25.1%)
No	155 (68.9%)	167 (74.9%)
Prior use of prescription drugs for recreational purposes (n, %)		
Yes	18 (8%)	18 (8.1%)
No	207 (92%)	205 (91.9%)

**Table 2:** Risk behaviors among patients who indicated history of sexual intercourse in military adolescent clinics (n = 315).

Variable	Active Duty (n = 196)	Dependent (n = 119)
Used a condom (self or partner) during most recent intercourse (n, %)		
Yes	113 (58.2%)	73 (67%)
No	81 (41.8%)	36 (33%)
Used any prescription birth control during most recent intercourse (n, %)		
Yes	112 (58.6%)	64 (59.8%)
No	79 (41.4%)	43 (40.2%)
Used any prescription birth control **and** a condom (self or partner) during most recent intercourse (n, %)		
Yes	76 (39.2%)	43 (39.4%)
No	118 (60.8%)	66 (60.6%)
Used any prescription birth control **or** a condom during most recent intercourse (n, %)		
Yes	149 (78%)	94 (87.9%)
No	42 (22%)	13 (12.1%)
Used alcohol or illicit drugs before most recent intercourse (n, %)		
Yes	32 (17.5%)	15 (14%)
No	151(82.5%)	92 (86%)
Reports history of chlamydia or gonorrhea infection (n, %):		
Yes	13 (6.6%)	15 (12.6%)
No	183 (93.4%)	104 (87.4%)

In the combined study population, 354 participants were between 17–23 years of age. Adolescents and young adults in the active duty group were more likely to be older (p = 0.001), male (p<0.001), White (p = 0.002), previous tobacco users (p = 0.008), and report a history of sexual activity (p = 0.001) as compared to the dependent group (Table 3). Of the 287 participants who reported previous sexual intercourse, the active duty group was less likely to use any birth control method (participant or partner) at their most recent intercourse (p = 0.049) (Table 4).

**Table 3:** Participant characteristics and risk behaviors among patients aged 17–23 who presented to military adolescent clinics (n = 354).

Variable	Active Duty (n = 225)	Dependent (n = 129)	P Value
Age (mean, SD)	20.3 (1.6)	18.8 (1.5)	**P = .001**
Gender (n, %)			
Male	129 (57.3%)	41 (31.8%)	**P < .001**
Female	96 (42.7%)	88 (68.2%)	
Race (n, %)			**P = .002**
White	137 (60.9%)	58 (45.0%)	
Black	33 (14.7%)	28 (21.7%)	
Hispanic	32 (14.2%)	35 (27.1%)	
Other	23 (10.2%)	8 (6.2%)	
Trouble paying household expenses (n, %)			**P < .001**
Yes	123 (54.7%)	38 (29.5%)	
No	102 (45.3%)	91 (70.5%)	
Ever had sexual intercourse (n, %)			**P < .001**
Yes	196 (87.1%)	91 (70.5%)	
No	29 (12.9%)	38 (29.5%)	
Smoked ≥ 100 cigarettes in lifetime (n, %)			**P = .008**
Yes	64 (28.4%)	21 (16.3%)	
No	161 (71.6%)	108 (83.7%)	
Over past 3 months, consumed alcohol-containing drinks at least weekly (n, %)			P = .53
Yes	23 (10.2%)	16 (12.4%)	
No	202 (89.8%)	113 (87.6%)	
Prior marijuana use (n, %)			P = .40
Yes	70 (31.1%)	47 (36.4%)	
No	155 (68.9%)	82 (63.6%)	
Prior use of prescription drugs for recreational purposes (n, %)			P = .18
Yes	18 (8%)	16 (12.4%)	
No	207 (92%)	113 (87.6%)	

Note: P Values calculated by Fisher Exact Test or Pearson’s Chi Square.

**Table 4:** Risk behaviors among patients aged 17–23 who indicated history of sexual intercourse in military adolescent clinics (n = 287).

Variable	Active Duty (n = 196)	Dependent (n = 91)	P Value
Used a condom (self or partner) during most recent intercourse (n, %)			P = .23
Yes	113 (54.2%)	56 (65.9%)	
No	81 (41.8%)	29 (34.1%)	
Used any prescription birth control during most recent intercourse (n, %)			P = .33
Yes	107 (57.5%)	53 (63.9%)	
No	79 (42.5%)	30 (36.1%)	
Used any prescription birth control **and** a condom (self or partner) during most recent intercourse (n, %)			P = .52
Yes	71 (37.6%)	35 (41.7%)	
No	118 (62.4%)	49 (58.3%)	
Used any prescription birth control **or** a condom during most recent intercourse (n, %)			**P = .049**
Yes	149 (78%)	74 (88.1%)	
No	42 (22%)	10 (11.9%)	
Used alcohol or illicit drugs before most recent intercourse (n, %)			P = .94
Yes	32 (17.5%)	15 (17.9%)	
No	151 (82.5%)	69 (82.1%)	
Reports history of chlamydia or gonorrhea infection (n, %)			**P = .017**
Yes	14 (7.4%)	15 (16.7%)	
No	176 (92.6%)	75 (83.3%)	

Note: P Values calculated by Fisher Exact Test or Pearson’s Chi Square.

**Fig 1:**
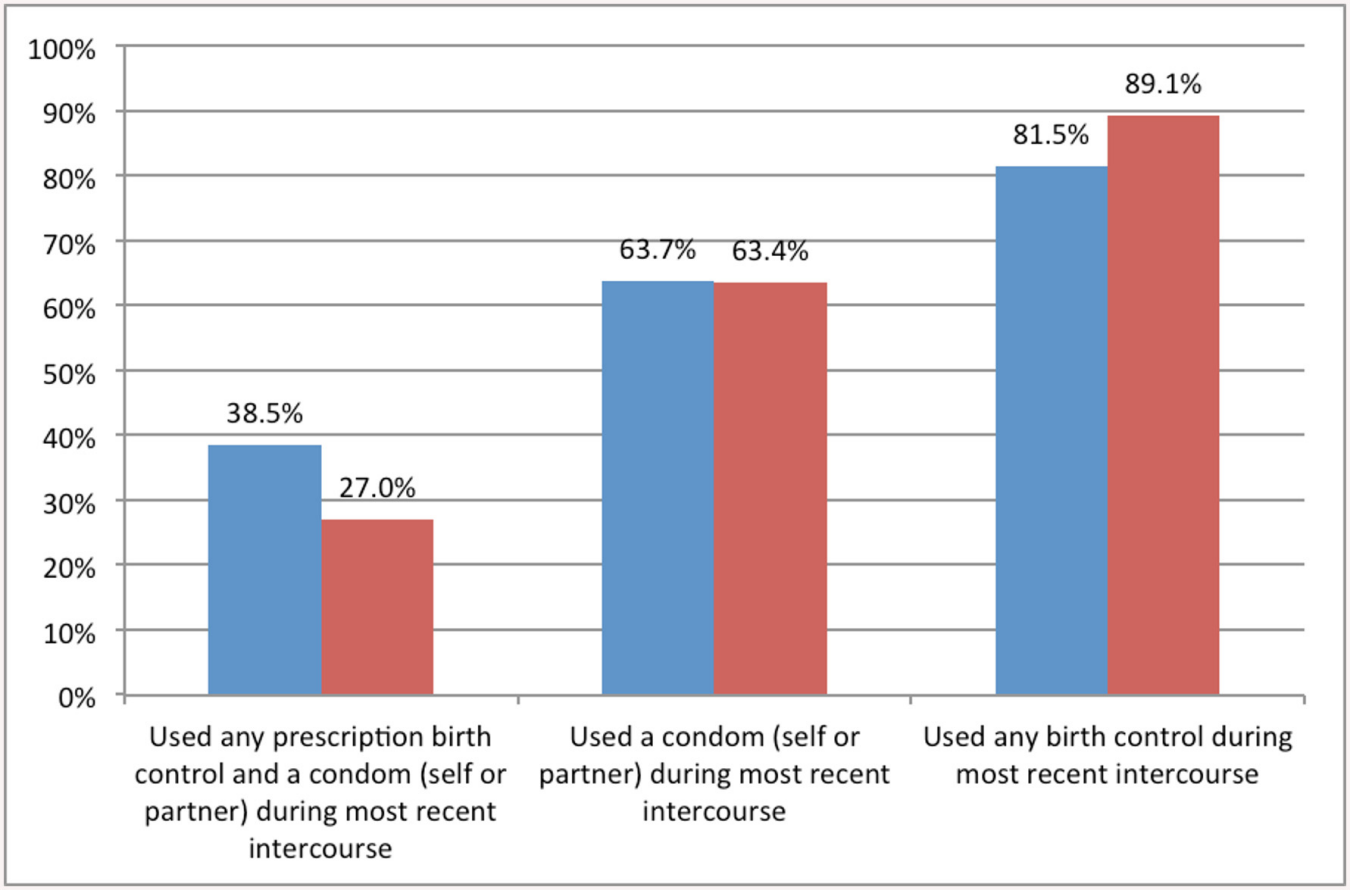
Use of contraception at last intercourse among participants aged 15–19: U.S. military population and National Survey of Family Growth. Blue Bars: Data from study population, n = 159. Red Bars: Data from National Survey of Family Growth 2006–2010, n = 6,145.

In total, 240 participants in the study population were between 15–19 years of age. Of these, 66.3% reported a history of sexual intercourse, 11.7% reported previous use of at least 100 cigarettes, and 24.6% reported prior marijuana use. Of the 159 participants who reported previous sexual intercourse, 56.6% used (or their partner used) prescription contraception, 63.7% used condoms, 81.5% used either method, and 38.5% used both methods at their most recent intercourse (Fig 1). In this age group, 9.7% reported prior Chlamydia Trachomatis or Neisseria Gonorrhoeae infection.

In bivariate analysis of all participants who reported prior intercourse, non-White participants reported less use of prescription birth control at most recent intercourse compared to White participants (p = .043), but not condom use (p = 0.751) or use of either method (p = 0.109). In logistic regression analysis, non-White participants were less likely to use either method as compared to White participants (OR = 0.447; 95% CI = 0.236, P = 0.846). Participants who reported attraction to the same or both genders were less likely to use either method compared to those who report attraction to the opposite gender (OR = 0.439; 95% CI = 0.198, 0.976) (Table 5). Age, gender, duty status, substance use at most recent intercourse, or previous marijuana or prescription drug use were not predictive of contraception use.

**Table 5:** Logistic regression analysis of prescription birth control or condom use during most recent intercourse among patients in military adolescent clinics.

	Sexually Active Participants N = 286^^^	
Variables	Odds ratio (95% C.I.)	p-value
Duty Status		0.081
Active Duty (reference)	1.0	
Dependent	2.107 (0.913, 4.860)	
Gender		0.744
Male (reference)	1.0	
Female	1.116 (0.578, 2.155)	
Ethnicity		**0.013**
White (reference)	**1.0**	
Not White	**0.447 (1.16, 4.1)**	
Sexual Attraction		**0.043**
Opposite Gender (reference)	**1.0**	
Same or Both Gender(s)	**0.439 (0.198, 0.976)**	
Substance Use Before Most Recent Intercourse		0.561
Yes (reference)	1.0	
No	0.771 (0.322, 1.850)	
History of Marijuana Use		0.845
Yes (reference)	1.0	
No	0.849 (0.422, 1.705)	
History of Recreational Prescription Drug Use		0.755
Yes (reference)	1.0	
No	0.617 (0.222, 1.715)	
Age (Continuous)	0.923 (0.764, 1.116)	0.408

^^^ 286 of the 315 patients who engaged in previous sexual activity had complete data.

* In this table, bold face numbers are statistically significant (P<0.05).

## Discussion

This is the first study that characterizes differences between patients presenting to military treatment facilities that predominately care for young active duty service members or military dependent adolescents within the same patient catchment area. Understanding these differences is important because they have direct implications for patient care and clinic resourcing. Adolescents in the active duty group were more likely to be older, male, White, previous tobacco users, report a history of sexually activity, and report less contraception use at their most recent intercourse, as compared to the dependent group.

These findings are consistent with what is known about the demographics of young military personnel and the differences between those who are self-sufficient as compared to those cared for by others. Although all military-affiliated youth should be screened for risk behavior, the young active duty service members may need additional attention as they transition to the work force and particularly one in which relocation, deployment, and work demands can be particularly stressful.

Approximately two-thirds of 15–19 year olds in the combined study population reported a history of sexual intercourse. This is higher than what has been found in samples of the national population. For example, the 2013 Youth Risk Behavioral Surveillance System (YRBSS)[24], which examined a national sample of high school students, suggests that almost half of high school students have engaged in sexual intercourse. Furthermore, the 2006–2010 National Survey on Family Growth (NSFG) found that 57% of adolescents in the United States have engaged in vaginal intercourse[25–26]. The high rates of reported intercourse in our study population may be due to the older age of our participants compared to the YRBSS, or the broad definition of intercourse used in our study design (i.e. the NSFG includes only vaginal intercourse). It is imprecise to compare the data from this study with the YRBSS or NSFG due to methodological and age differences; however, it appears that military youth may experience a relatively high rate of intercourse compared to their civilian counterparts (Fig 1).

In the United States, 59% of high school students report use (or partner use) of condoms, 25% report use of prescription birth control, 9% report use of condoms plus prescription birth control, 14% report having used no birth control at the most recent sexual intercourse, and 22% report use of alcohol or drugs before most recent intercourse[24]. The higher rate of prescription contraceptive use in the military population may be due to their receipt of universal healthcare.

One-quarter of the 15–19 year olds in the study population reported previous marijuana use, and 10% have used prescription drugs for recreational purposes. Nationally, 41% have tried marijuana and 18% have used prescription drugs for recreational purposes[24]. While rates of substance use in the military population may be lower than national rates, they are substantial. Therefore, clinicians caring for military affiliated adolescents should screen for sexual behavior and substance use, and provide appropriate counseling, contraception, substance abuse treatment, and screening for associated infections.

Racial disparities in contraception use at most recent intercourse found in logistic regression analysis appear to be the result of prescription contraceptive use as opposed to condom use in the study population. This finding is consistent with the results of a large population-based study in the United States, which suggested that 85% of 15–19 year olds used some form of contraception (e.g. condoms and/or prescription contraceptives) at their most recent intercourse, and White adolescents were approximately twice as likely as Hispanic and Black adolescents to use common prescription contraceptives[25,27].

To our knowledge, this study is among the first that specifically inquired about sexual preference among active duty military service members since the repeal of the “Don’t Ask, Don’t Tell” legislation in 2011. Over 10% of the participants in our study indicated attraction to members of the same gender or both genders. These results are consistent with a recent study of individuals located in the United states, suggesting that approximately 4 percent of Americans report that they identify as lesbian, gay, bisexual or transgender, 8 percent report that they have engaged in same-sex behavior, and 11 percent acknowledged some same-sex attraction[28].

Clinicians caring for military-affiliated adolescents and young adults should be well trained in caring for persons who identify as being a sexual minority. Most adolescents warrant routine screening for Chlamydia Trachomatis, Neisseria Gonorrhoeae, Human Immunodeficiency Virus, and Syphilis; and primary prevention with immunizations covering for Hepatitis A and B and Human Papillomavirus Viruses, and sexuality education regarding such infections[29]. Men who have sex with men benefit from more frequent and more robust screening for sexually transmitted infections (e.g. oral/anal sites; hepatitis B testing)[21,29]. Women who have sex with women should undergo education and testing with respect to their level of risk (e.g. cervical cancer screening per national guidelines; STI screening; and education about contraception and spread of infection as indicated)[21,29]. Care of such individuals should be performed by clinicians without judgment, to foster open communication, accurate risk assessment, and therapeutic relationships[21,29].

There are limitations to this study. Since the data collection was cross-sectional, causality cannot be determined. There was no data collected on military officers; accordingly, the study may not be representative of that population. Convenience sampling was used in the clinic setting. Therefore, the population studied may be at higher risk than the general military population. However, this limitation was partially attenuated because participants were recruited consecutively and few did not agree to participate. This study was designed to be exploratory in nature.

Based on the findings of this study, adolescents and young adults seen in military clinics engage in high-risk sexual behavior and substance use. Furthermore, approximately 10% of this population expresses same-sex attraction. Due to these patient factors, clinicians who care for military youth should be comfortable assessing their patient’s psychosocial history and providing appropriate clinical recommendations. Further study is warranted to identify psychosocial factors that may influence risk and resilience among young military healthcare beneficiaries. The extent to which healthcare disparities exist within this healthcare system needs additional examination.

## Military Clause

The view(s) expressed herein are those of the authors and do not reflect the official policy or position of the U.S. Army Medical Department, the U.S. Air Force Medical Department, the Department of the Army, the Department of the Air Force, the Department of Defense, or the U.S. Government.
